# God Helps Those Who Help Themselves: How Recipients’ Efforts Perception Affects Donation

**DOI:** 10.3389/fpsyg.2021.695332

**Published:** 2021-10-06

**Authors:** Fei Jin, Zhengyu Zheng, Banggang Wu

**Affiliations:** Department of Marketing and E-Commerce, Business School, Sichuan University, Chengdu, China

**Keywords:** recipients’ efforts, empathy, need severity, donation, helping

## Abstract

This research examines how recipients’ efforts to get out of the plight affect the empathy they evoke and the subsequent help they receive from donors. Through three experiments, we find that the higher the efforts made by the recipients to get out of the plight, the stronger the donors’ willingness to donate. This effect is moderated by the need severity of the recipients. The more serious the plight is, the stronger the impact of the degree of efforts on the willingness to donate. This research makes theoretical contributions to charitable giving and provides implications for non-profit organizations on describing recipients’ efforts to get out of their plight.

## Introduction

With an increasing number of charitable organizations, individual donations play an import role. According to Giving USA 2021, individuals gave $324.10 billion and represented 69% of all charitable giving in 2020. Faced with various donation requests from charities, consumers with limited financial resources have to select which one to support. Numerous charities, such as the Red Cross organization, raises funds for recipients who may put great efforts (hereafter we use recipients’ efforts to refer to perceptions of whether recipients try to improve the situation) to get out of the plight. For instance, many fundraisers try to attract attention through framing the recipient as a person with spirit of self-improvement and succeed in increasing donations. There are other recipients who are passively waiting supports from others (Weiner et al., 1988). Which recipients are more likely to get help? How does recipients’ efforts to improve the situation influence donation decisions of consumers?

Previous research has focused on examining how to attract supports through designing donation appeals (e.g., Ajzen et al., 2000; White and Peloza, 2009; Kristofferson et al., 2014) as well as individual donor characteristics inspiring consumer donations (Reed et al., 2007; Winterich et al., 2012; Erlandsson et al., 2020). However, most of this research has focused on support for recipients that deserve help without taking the recipients’ own behavior in to consideration. One stream of research considering recipients tends to focus on the number of recipients (e.g., a single identifiable victim, victims that are perceived as entitative; Kogut and Ritov, 2007; Smith et al., 2013) or by group membership (Duclos and Barasch, 2014; Touré-Tillery and Fishbach, 2017). However, as people have limited financial resources, they may evaluate the whether the recipients deserve help (Bekkers and Wiepking, 2011) and whether they have the responsibility to help (Erlandsson et al., 2015) before making donations. For instance, when the victim is perceived responsible for his plight, identifiability decreases helping mostly when people hold a strong belief in a just world for others (Kogut, 2011). This research examines when and why consumers engage in charitable giving by considering the characteristics of the charity recipients.

We theorize and empirically demonstrate that recipients’ efforts to improve the situation will increase people’s empathy and thus boost willingness to donate. Moreover, the effect of the degree of effort on the willingness to donate increases the positive effect of recipients’ efforts on empathy and helping is aggravated when the recipients are in severe need such that they are in obvious distress. One thing to merit, the current research focuses specifically on the recipients’ efforts to get out of a miserable situation, but not his/her faults to be there (Engel, 2011). We do not mention if recipients are responsible for getting into the plight.

Our research extends existing work on charitable donation (White and Peloza, 2009; Winterich et al., 2013; Erlandsson et al., 2020) by shedding light on fact that recipients should be considered as an important factor influencing consumers’ donation decisions. We also contribute to the empathy literature by exploring the underlying mechanism. Specifically, we demonstrate a novel means of evoking empathy—recipients’ efforts and this effect is more salient when the need is severe.

## Theoretical Framework

### Recipients’ Efforts and Donation

The goal of donations is to help those in need get out of troubles and improve their lives. The premise of donation is that people in need of help from others are unable to get out of trouble on their own and need helps from others (Bendapudi et al., 1996). When deciding whether to donate or not, donors measure their costs and benefits, that is, whether their good deeds can bring some benefits to themselves or others, which is similar to the concept of “value” (deservedness) in traditional Chinese culture. When deciding to help others, donors will inevitably consider whether this person is worth helping and tend to seek cues from recipients’ characteristics and behavior, one of which is whether he has made efforts to improve the plight. For example, some people are reluctant to help young people begging at the train station because they do not try their best to improve their lives and rely entirely on the help from others. Previous research also found that people are more likely to help recipients in naturally caused rather than humanly caused disasters. This is driven by a perception that the victims of natural disasters are to be blamed less for their plight, and that they make more efforts to help themselves (Zagefka et al., 2011). As a result, the level of efforts of those in need has a direct impact on the willingness to contribute (Barnes et al., 1979). On the other hand, if the plight does not get improved even when the people in need try their best to improve the plight. It means that it is difficult to improve on their own, and victims really need the help from outside. In this context, donors also see the value of their contributions, thereby increasing their willingness to contribute.

Based on empathy, donors experience the difficulties of the people in need, thus reflecting a higher willingness to donate (Fisher et al., 2008). De Waal (2008) puts forward three conditions that enhance empathy: First, people must identify with others and are willing to think from the perspective of each other. Second, people can evaluate the causes of specific emotions. Third, people are affected by the feelings of the victims and then produce the same feelings. These three conditions are the basic conditions for the formation of empathy, which complement each other.

The level of recipients’ efforts of those in need will increase the empathy of donors for the following reasons: first, the victims have tried their best to improve their plight, but if the plight still exists, it means that the recipients are in great difficulties, and this difficult situation will arouse the empathy of donors. Second, the spirit of those in need who first rely on their own efforts to change the plight can also easily move donors and inspire their strong empathy. Extant research has shown that empathy accompanies treatment of “deserving” poor people, and anger typically accompanies treatment of “undeserving” poor people. Hence, people are not willing to help recipients eliciting anger (Small and Lerner, 2008). As a result, the greater the efforts made by those in need to improve their plight, the higher the level of empathy among donors. Put it formally,

H1: People are more likely to donate when recipients’ efforts are high (vs. low).

### The Mediating Roles of Empathy

The empathy-helping hypothesis specifies that people are most likely to help when they vicariously experience the distress of those in need (Batson, 1987). Eisenberg and Miller (1987) refer to empathy as an emotional state derived from understanding others’ emotional state. De Waal (2008) demonstrates that there are three criteria for empathy: “Empathy is the capacity to (1) be affected by and share the emotional state of another, (2) assess the reasons for the other’s state, and (3) identify with the other, adopting his or her perspective.”

We suggest that recipients’ efforts will increase the empathy of donors for the following reasons. First, the victims have tried their best to improve their plight, but if the plight still exists, it means that the victims do face great difficulties and this difficult situation will arouse the empathy of donors. Second, donors are more easily to touched by the spirit of those in need who first rely on their own efforts. Studies have shown that empathy motivates helping behavior (Fisher et al., 2008; Fisher and Yu, 2014; Lee et al., 2014). It is because empathy has prompted donors to experience their difficulties from the perspective of the victims, which leads to great psychological discomfort among donors, stimulating their desire to help recipients out of trouble (Fisher and Yu, 2014).

Previous research has demonstrated that perceived impact of helping also has an important influence on donations. For example, research shows people are more responsive to a charitable solicitation when informed that a third party will match their gift amount (Karlan and List, 2007). However, this perceived impact-based proposition makes no predictions about the role of empathy in motivating donations. The present research explores the emotional effects of recipients’ efforts to improve the situation on empathy, and the resulting helping tendencies. Hence,

H2: Empathy mediates the effect the recipients’ efforts and donation.

### The Moderating Role of Need Severity

Donations are more necessary when the recipient is in obvious need. The level of empathy that is produced in observers changes with the victim’s need. For instance, a person without any other supports naturally evokes more empathy than a person whose family can provide partial of the care he/she requires. The greater the victim’s suffering, the greater the vicarious pain experienced by observers and the stronger their feelings of sympathy for the victim. In this study, we further propose need severity, which we define a severe need as a state that is with either a high level of immediate suffering or a vulnerability to future harm, moderates the relationship between recipients’ efforts and donation. In fact, previous research has shown that the more difficult it is for recipients, the easier it is to get help from others (Bendapudi et al., 1996; Batson et al., 2005). Given the important role of the victim’s need in eliciting empathy, how does recipients’ efforts to improve the situation affect the degree to which they are perceived to be needy? We propose that recipients’ efforts increase helping when the victim’s need is obviously severe. First, if the plight is very serious, it means that there is a high urgency to improve the situation. At this situation, donors’ empathy can hardly be motivated if victims themselves do not work hard to improve the plight. Second, if donors perceive that the victim is in a very difficult situation, but the victim does not make corresponding efforts to improve his/her situation, this may give the donor the feeling that the victim does not want to improve the plight so much. Since the recipient does not have a strong desire to make a change, how can the donor have the incentive to initiate helping?

One could argue that people will also help the undeserving (i.e., those who do not put efforts to improve the situation) if recipients will die without help. We admit this maybe the case. However, we do not focus on this extreme situation in this research. Another possibility is that people will help those who put efforts than those do not put efforts when the situation is not that severe. Because there is a chance for those who do not put efforts to “learn a lesson” that they would make a greater effort to help themselves next time. Note that this account is different from our empathy explanation in that it focuses on a future orientation that people assume the recipients will change their behavior. In this research, what we want to show is that people feel more compelled to help by high levels of sympathy, when recipients put great efforts to improve the situation (vs. not). Put it in formal,

H3: Need severity of the plight aggravates the effect of recipients’ efforts on donation.

## Study Overview

Three experiments were designed to examine the proposed hypotheses. Study 1 showed initial evidence that people would like to donate to those who put efforts in the plight. Study 2 replicated the effect in a different scenario and demonstrated that empathy mediated the effect. Moreover, we ruled out the alternative explanation of expected impact. Study 3 was a lab study with real donation behavior. It extended the previous studies by documenting the moderating role of need severity. People were more likely to donate to those who put efforts in the high severity condition (vs. low severity condition). In sum, across three studies using different scenarios and samples, we provided consistent evidence showing when and why recipients’ efforts increase donors’ prosocial behavior.

## Study 1 Main Effect of Efforts

The main purpose of this study was to explore the impact of recipients’ efforts on people’s willingness to donate. According to the hypotheses, the greater the efforts made by the people in need to improve their plight, the more likely people will donate to them.

### Procedure

One hundred twenty participants (M_*age*_ = 40.9 years, *SD* = 11.5; 56.7% female) from Amazon Mechanical Turk participated in exchange for monetary compensation ($0.25). They were randomly assigned to two groups (high efforts vs. low efforts).

We told them that a U.S. charity called Hope for Tomorrow Foundation is raising money to help children who do not have enough financial support to pursue education. To rule out the potential influence of the reasons for getting into trouble, we told participants that recipients in this experiment are in trouble because of external reasons. In the high efforts condition, the boy named Jim tries his best to read books and learn from any possible ways. As long as he has a little spare money, he will use it to buy books. In the low efforts condition, the boy named Jim will read books when his parents ask him to do so. He likes playing outside.

Then we asked their willingness to donate to Jim (1 = not at all, 7 = very much). We also asked how much they would like to donate to Jim if they had 50 dollars to allocate (from 0 to 50).

After that, we asked their perception of recipient efforts which served as the recipients’ efforts manipulation check (To what extent do you think Jim is striving for tomorrow? To what extent do you think Jim put effort in changing *status quo*? *r* = 0.79).

Participants then indicated age and gender. Finally, they were debriefed.

### Results and Discussion

#### Manipulation Check

The results of one-way ANOVA showed that participants in the high efforts condition thought that Jim put greater efforts (*M* = 5.02, *SD* = 1.24) than those in the low efforts condition [*M* = 3.20, *SD* = 1.70; *F*(1, 118) = 44.83, *p* < 0.01].

#### Donation Intention

As expected, participants had higher intention to donate when they found that the recipient made a large effort to help himself (*M* = 4.43, *SD* = 1.52) rather than low efforts [*M* = 3.77, *SD* = 1.58; *F*(1, 118) = 5.55, *p* = 0.02].

#### Money Donation

The results of one-way ANOVA showed that participants donated more in the high efforts condition (*M* = 17.35, *SD* = 6.17) than in the low efforts condition [*M* = 12.53, *SD* = 6.17; *F*(1, 118) = 7.74, *p* < 0.01].

Study 1 showed initial evidence that the extent of the efforts of those in need affects donors’ intention to contribute. People are more willing to help those in need who put efforts to improve their situation.

## Study 2 the Mediating Role of Empathy

In Study 2, we aimed to test the mediating role of empathy on the relationship between recipients’ efforts and willingness to donate. We predicted that when recipients’ efforts were high, people would be more likely to donate. More importantly, we predicted that empathy would mediate the proposed effect.

### Procedure

One hundred twenty-four participants (M_*age*_ = 31.4 years, *SD* = 8.7; 36.3% female) recruited through Amazon Mechanical Turk completed this study in return for monetary compensation ($0.30). They were randomly assigned to a 2 (high efforts vs. low efforts) between-subjects design. Participants read a message from a fictitious organization called Children Care. The organization was holding a campaign for children with hearing impairment. In the high efforts condition, we told participants that these children learned to be a normal individual and do exercise every day to get their hearing back. The process was arduous but they never gave up. In the low efforts condition, participants read that these children learned to be a normal individual and do exercise to get their hearing back. Right after the description, there was a slogan in high efforts (low efforts) condition: Help those who make an effort! (Help those who are in need!).

#### Empathy

Empathy was measured in terms of the empathic emotions elicited by a victim’s suffering (Batson, 1991; Batson et al., 2005). We asked participants to report the degree to which they felt sympathetic, compassionate, softhearted, warm, and moved in response to the child’s situation on a seven-point scale (1 = not at all, 7 = extremely; α = 0.86).

#### Expected Impact

Next, participants answered three questions measuring the extent to which they felt they could have an influence on the issue by donation (Touré-Tillery and Fishbach, 2017). We asked them to indicate how much impact they would expect to make to this organization, to what extent they would expect their donation to advance the work of this organization and how much they would expect their donation to benefit the recipients (1 = Not at all, 7 = Very much; α = 0.91).

We then measured prosocial intention by asking participants how much they would be willing to donate to Children Care to support the campaign. We also asked them if they would like to share this campaign on their social media to ask more people involve in it (1 = Yes, 0 = No).

After that, we asked their perception of recipient efforts as the same in Study 1 (*r* = 0.81). Participants then indicated age and gender. Finally, we debriefed participants.

### Results and Discussion

#### Manipulation Check

The results of one-way ANOVA showed that participants in the high efforts condition perceived greater efforts (*M* = 5.27, *SD* = 1.06) than those in the low efforts condition [*M* = 4.16, *SD* = 1.96; *F*(1, 122) = 15.47, *p* < 0.01].

#### Donation Intention

As expected, participants would be more likely to donate when they found that the recipient made a large effort to help himself (*M* = 5.37, *SD* = 1.26) rather than low efforts [*M* = 4.31, *SD* = 2.14; *F*(1, 122) = 11.41, *p* < 0.01].

#### Whether to Share

The results of chi-square showed that participants were more likely to share this campaign on social media when they thought that recipients put high efforts than low efforts (67.7 vs. 32.3%; Wald = 4.77, *p* = 0.03).

#### Empathy

The results of one-way ANOVA showed that participants in the high efforts condition showed more empathy to the recipients (*M* = 4.56, *SD* = 1.74) than those in the low efforts condition [*M* = 3.76, *SD* = 1.72; *F*(1, 122) = 6.75, *p* = 0.01].

#### Expected Impact

Results showed participants believed their donation would not have significant impact when recipients were in high efforts (*M* = 4.42, *SD* = 1.80) than they were in the low efforts [*M* = 4.06, *SD* = 1.79; *F*(1, 122) = 1.21, *p* = 0.27].

#### Mediation Analysis

**FIGURE 1:**
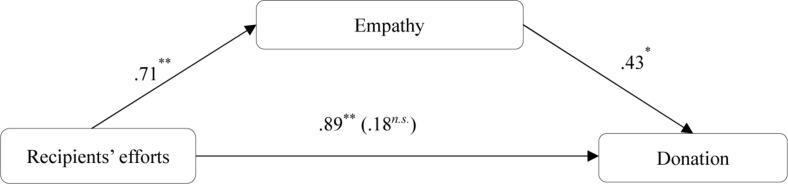
Mediation effect of empathy, Study 2. *p < 0.05, **p < 0.01.

We predicted recipients’ efforts would influence donation intention, at least in part, through empathy. To test this proposed underlying mechanism, we conducted a mediation analysis (Preacher et al., 2007; SPSS Process Macro Model 4) using bootstrapping procedures (*N* = 5,000). We put both empathy and expected impact into the process. Results showed that recipients’ efforts predicted the empathy [β = 0.71, *SE* = 0.22, *t*(122) = 2.69, *p* < 0.001]. Next, a regression that included recipients’ efforts and empathy revealed that empathy significantly predicted donation intention [β = 0.43, *SE* = 0.23, *t*(122) = 2.68, *p* = 0.03], while recipients’ efforts no longer predicted donation intention [β = 0.18, *SE* = 0.03, *t*(122) = 0.01, *p* = 0.08]. Finally, the 95% bias-corrected confidence interval did not include 0 [95% CI (0.016, 0.378); Figure 1]. While for the expected impact, we only found that it positively affected donation [β = 0.23, *SE* = 0.05, *t*(122) = 0.65, *p* = 0.02]. But the 95% bias-corrected confidence interval included 0 [95% CI (−0.044, 0.324)], suggesting that expected impact did not mediate the proposed effect.

Overall, Study 2 showed further evidence that people would like to donate when they perceive that recipients are in high efforts. Moreover, we found that empathy mediates the relationship between recipients’ efforts and donation intention.

## Study 3 the Moderating Role of Need Severity

Study 3 was a real donation behavior study and it had two goals. First, we aimed to provide consistent evidence that recipients’ efforts increase donation intention in a different scenario. Second, we investigated our process explanation with a test of the role of perceived need as moderator.

### Procedure

One hundred fifteen participants (M_*age*_ = 21.7 years, *SD* = 1.7; 48.7% female) from a university in China came to the lab to participate this experiment in exchange for 10 RMB. They were randomly assigned to a 2 (high efforts vs. low efforts) × 2 (high need severity vs. low need severity) between-subjects design.

At the beginning of the study session, we gave participants two white envelopes. One of the envelopes was labeled “Compensation” and contained their compensation for participating in the study. The other envelope was labeled “Donation.” We told participants that they would get 10 RMB, so that they could allocate any amount ranging from 0 to 10 RMB as a donation (Lee and Shrum, 2012). We would donate these incomes to the charity when the experiment finished.

Participants were then shown a description from the Green Ribbon: “This organization provides medical treatment for people who are unable to afford the necessary medical treatment for serious diseases. Most of the patients do not have medical insurance because they do not hold a steady job.”

#### Recipients’ Efforts

In the high efforts condition, participants were told that a girl of thirty tried her best to work, whereas in the low efforts condition, they were told that a girl of thirty gave up work and waited for regular donations from the charity.

#### Need Severity

In the high-need severity condition, participants were told that the girl’s house was destroyed and her parents had died. In the low-severity condition, participants were told that the girl had a big family.

After reading the description, participants indicated their donation likelihood, empathy toward the recipients. Participants then indicated age and gender. Finally, we debriefed participants.

### Results and Discussion

#### Manipulation Check

The manipulation check of recipients’ efforts was the same with Study 1 and 2 (*r* = 0.80). The efforts manipulation had the expected effect on perceived efforts [*F*(1, 114) = 12.32, *p* < 0.01], that participants in the high efforts condition had higher efforts perception (*M* = 4.43, *SD* = 1.58) than those in the low efforts condition (*M* = 3.46, *SD* = 1.38).

The manipulation of need severity was successful [*F*(1, 114) = 17.17, *p* < 0.01] that participants perceived more severity in the high severity condition [*M* = 4.87, *SD* = 1.26] than in the low severity condition (*M* = 3.74, *SD* = 1.62).

#### Likelihood to Donate

**FIGURE 2:**
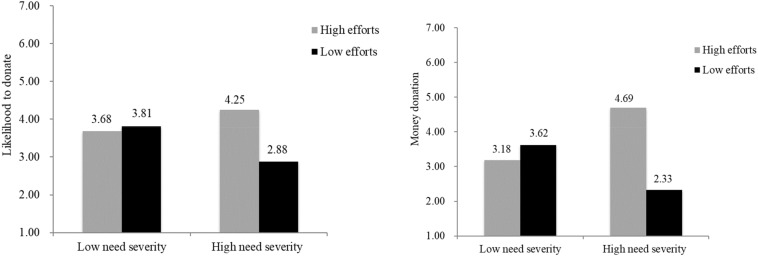
Likelihood to donate and money donation, Study 3.

Results of the two-way ANOVA supported our hypotheses. It revealed a significant interaction between recipients’ efforts and need severity on likelihood to donate [*F*(1, 111) = 7.05, *p* < 0.01] and main effect of recipients’ efforts [*F*(1, 111) = 4.84, *p* = 0.03]. But there was no significant main effect of need severity [*F*(1, 11) = 0.42, *p* = 0.52]. Simple effects tests indicated that participants in the low need severity condition did not show different likelihood to donate in the low recipients’ efforts (*M* = 3.81, *SD* = 1.60) or high recipients’ efforts [*M* = 3.68, *SD* = 1.78; *F*(1, 111) = 0.10, *p* = 0.75]. In the high need severity condition, participants were more likely to donate when recipients put efforts (*M* = 4.25, *SD* = 1.27) than those do not put efforts [*M* = 2.88, *SD* = 1.26; *F*(1, 111) = 11.74, *p* < 0.01; Figure 2].

#### Money Donation

We also checked participants’ money donation behavior. Results of the two-way ANOVA showed a significant interaction between recipients’ efforts and need severity on money donation [*F*(1, 111) = 12.415, *p* < 0.01] and main effect of recipients’ efforts [*F*(1, 111) = 5.83, *p* = 0.02]. But there was no significant main effect of need severity [*F*(1, 111) = 0.08, *p* = 0.79]. Simple effects tests indicated that for participants in the low need severity condition, the amount of money donated did not have significant differences in the low recipients’ efforts (*M* = 3.62, *SD* = 2.25) or high recipients’ efforts [*M* = 3.18, *SD* = 1.10; *F*(1, 111) = 0.62, *p* = 0.43]. However, in the high need severity condition, participants were more likely to donate when recipients put high efforts (*M* = 4.69, *SD* = 2.75) than those in the low efforts condition [*M* = 2.33, *SD* = 1.27; *F*(1, 111) = 17.57, *p* < 0.01].

#### Moderated Mediation

Our theory predicts that empathy mediated the effect of recipients’ efforts on donation when need severity was high but not when it is low. Following the procedure recommended by Zhao et al. (2010), mediation is established if the indirect effects of the independent variables on the dependent variable are significant. We used bootstrapping to compute the 95% CI of the conditional indirect effect for recipients’ efforts (Preacher and Hayes, 2004) when severity is low and when it is high, respectively. The results showed that the mediation results only held when severity is high [95% CI (0.042, 0.143)].

In summary, we found support for H3 using actual donation behavior that recipients’ efforts have a positive effect on donation intention for children who are in the high need severity condition.

## General Discussion

Consumers have limited financial resources to donate, and thus charities compete to attract donors’ support. This research builds on the growing literature on charitable giving by extending from the previous “backward perspective” (i.e., whether recipients are responsible for the plight) to the “looking forward perspective” (i.e., whether recipients should be responsible for getting out of the plight). Across three studies, we show that recipients’ efforts lead to greater donations to beneficiaries (Study 1). The greater the efforts made by the recipients to get out of the plight, the more likely they can boost empathy (Study 2). Moreover, when recipients are in high (vs. low) need severity, donors value the efforts more, resulting in more empathy (Study 3). This finding was robust across different scenarios, different samples as well as an experiment with real monetary contributions in the lab.

### Theoretical Contribution

These findings add to the literature on charitable giving, empathy, as well as effects of perceived efforts. Despite the growing body of work on charitable giving in consumer research (Zagefka et al., 2011; Duclos and Barasch, 2014; Wang et al., 2021), only limited research has focused on characteristics of the recipients (Smith et al., 2013). Some of these studies found that people are less willing to provide support or aid to those in need when recipients are judged as personally responsible for their problems (e.g., Farwell and Weiner, 2000; Henry et al., 2004). Our results contribute to the literature on charitable giving by identifying another characteristic of recipients- their efforts to improve the situation as a factor that can increase donations to beneficiaries. Specifically, we show that donors have higher willingness to donate when they find the recipients put efforts to improve the situation.

Secondly, the theorizing and empirical demonstration of the mechanism underlying the effect of recipients’ efforts to improve the situation makes contribution to the literature in empathy. We demonstrate that empathy underlies the proposed effect. At a broader level, our findings that recipients’ efforts to improve the situation can elicit empathy contributes to the literature related to the importance of affect (vs. cognitions) in consumers’ charitable giving (e.g., Fisher et al., 2008; Small and Verrochi, 2009).

We further find that the negative effect of recipients’ efforts to improve the situation on empathy and helping is strengthened when recipients are in severe need. This is in line with previous research showing that when a child is in severe need, people are more likely to help based on on an emotional response to his or her suffering rather (Loewenstein and Small, 2007).

### Management Implication

From a managerial and societal perspective, this research may help non-profit organizations at large. With the increase of relief agencies and non-profit organizations, how to inspire donation from the public has become a concern. To solve this problem effectively, fundraisers must first realize that recipients, as the object of fundraiser marketing, has an important impact on attracting donors’ support.

Our research provides non-profit organizations with specific guidance on how to communicate recipients’ stories on charity websites, advertising, brochures, and other promotional materials. Our research shows recipients’ efforts can have a positive effect on empathy and thus increase donation. In practice, charities might benefit from selecting those who put great efforts to improve the situation. This objective might be achieved by varying the type of lens used, the angle of the photograph, and lighting conditions.

Second, we show that the degree of efforts to improve the plight made by recipients has a significant impact on donors’ empathy. Fundraisers tend to focus on the extent of recipients’ difficulties when designing donation appeals, emphasizing how “how pitiful” they are, in the hope of arousing public empathy. Our research suggests that charities can underline great qualities of recipients, such as self-improvement, self-reliance, optimism in the plight and so on. It is easy to win likes and recognition from the public.

Another important practical implication of our findings is that emphasizing the severity of the plight increases helping if the person in need make an effort to help oneself, but might even reduce helping if the person does not make an effort to help oneself.

### Limitations and Future Research Directions

Although this paper enriches the literature in the field of donation by exploring the impact of recipients’ efforts, there are still many aspects that could be improved in the future. First, the basic assumption of this study is that the recipients themselves can make corresponding efforts, but in some cases, the people in need may have lost the ability to do so. Therefore, future research can distinguish between those who are *unable* to make efforts and are *unwilling* to make efforts, and examine the differential impacts on the willingness to donate. A related question is: is portraying recipients’ efforts a good strategy to motivate donations? We propose that this is not always the case and depends on how donors infer these efforts. Showing the information that recipients put efforts to improve the situation helps when others do not know that the recipients try best to get out of plight. However, giving these information does not help, even hurts when others are already aware that the recipients put great efforts to improve the situation. Second, this study mainly explores monetary contributions. Past research has suggested that consumers have fundamentally different responses to money vs. time donation (Liu and Aaker, 2008; Macdonnell and White, 2015), future research could explore how recipients’ efforts influence donors’ time contribution. Third, it would be useful for future research to explore the potential for a non-linear relationship between recipients’ efforts and empathy. It is possible that the increase in empathy we observed only occurs when a recipient is perceived to be in a moderate effort level. Further, human judgments of efforts are subjective, so the effects we found are likely dependent on relative rather than absolute standards of efforts. Forth, we acknowledge that empathy is not the only mediator that explains the relationship between recipients’ efforts and donation. Future research can explore more potential underlying mechanisms.

## Data Availability Statement

The raw data supporting the conclusions of this article will be made available by the authors, without undue reservation.

## Ethics Statement

Ethical review and approval was not required for the study on human participants in accordance with the local legislation and institutional requirements. Written informed consent for participation was not required for this study in accordance with the national legislation and the institutional requirements.

## Author Contributions

FJ designed the research framework. ZZ carried out the survey. BW analyzed the data. FJ and BW wrote the manuscript. All authors contributed to the article and approved the submitted version.

## Conflict of Interest

The authors declare that the research was conducted in the absence of any commercial or financial relationships that could be construed as a potential conflict of interest.

## Publisher’s Note

All claims expressed in this article are solely those of the authors and do not necessarily represent those of their affiliated organizations, or those of the publisher, the editors and the reviewers. Any product that may be evaluated in this article, or claim that may be made by its manufacturer, is not guaranteed or endorsed by the publisher.
